# Self-Poisoning With Household Bleach in an Elderly Man

**DOI:** 10.7759/cureus.34957

**Published:** 2023-02-14

**Authors:** Rute Brás Cruz, Filipa David, Diana L Rocha, Adelina Pereira, Ernestina Gomes

**Affiliations:** 1 Internal Medicine, Hospital Pedro Hispano, Matosinhos Local Health Unit, Matosinhos, PRT; 2 Intensive Care Unit, Hospital Pedro Hispano, Matosinhos Local Health Unit, Matosinhos, PRT

**Keywords:** self-poisoning in the elderly, sodium hypochlorite, household bleach, self-poisoning, voluntary ingestion

## Abstract

Caustic self-poisoning is a major health hazard, which affects any age, but is particularly serious among the elderly. Household bleach is a caustic that contains 3% to 6% sodium hypochlorite solution, one of the most common agents in unintentional caustic poisoning. In this clinical case, we present a household bleach self-poisoning by an older man with no relevant medical history. He presented with extensive burns on the oral cavity mucosa and tongue, requiring orotracheal intubation by video laryngoscopy. He was then admitted to the intensive care unit, where he evolved poorly. Given the poor prognosis and the lack of physiological reserve for the invasiveness required for a surgical approach, a conservative strategy was chosen after a multidisciplinary team discussion. With the conservative strategy, the patient survived, being discharged to the general ward after one month, where he underwent a Stamm gastrostomy and placement of a percutaneous endoscopic gastrostomy. In the follow-up consultation three months later, the patient was found to be weakened, with high frailty status, presenting anxiety, depression, and causing high family burden.

## Introduction

Any alkaline agent with a pH greater than 7.0 is considered caustic. Bleach is a household caustic (pH 11) containing 3% to 5% sodium hypochlorite [1] and is one of the agents most commonly involved in caustic self-poisoning.

Caustic ingestion remains a significant global health hazard, despite ongoing educational programs and legislation on the concentration and availability of corrosive substances [2]. Contact of a caustic agent with any biological tissue can potentially cause cell death by denaturation [3] of protein and saponification [1] of lipids. Thrombosis of the microvasculature with progression to liquefaction necrosis has also been described [1]. In case of ingestion, it can cause esophageal and gastric injuries leading to esophageal or gastric perforation in severe cases. Other symptoms include secondary vomiting, aspiration pneumonitis, and pulmonary irritation due to the production of chlorine in the stomach [4]. This medical emergency can result in extensive morbidity and a high mortality rate [5].

Ingestions of caustic agents by children are usually accidental and harmless, given the small amounts ingested [6]. In contrast, ingestion in adults is usually intentional, involving larger amounts with more severe sequelae [6]. In the elderly, most cases are unintentional. They may result from product misuse, improper storage, misidentification, or confusion [7], leading to even more severe complications due to their comorbidities and high frailty, in contrast to younger groups [7].

Although ingestion of common bleach (sodium hypochlorite or hydrogen peroxide) is rarely associated with severe damage to the upper gastrointestinal tract [8]. We present a case of an older man with severe burns after an intentional ingestion of household bleach.

## Case presentation

**Figure 1:**
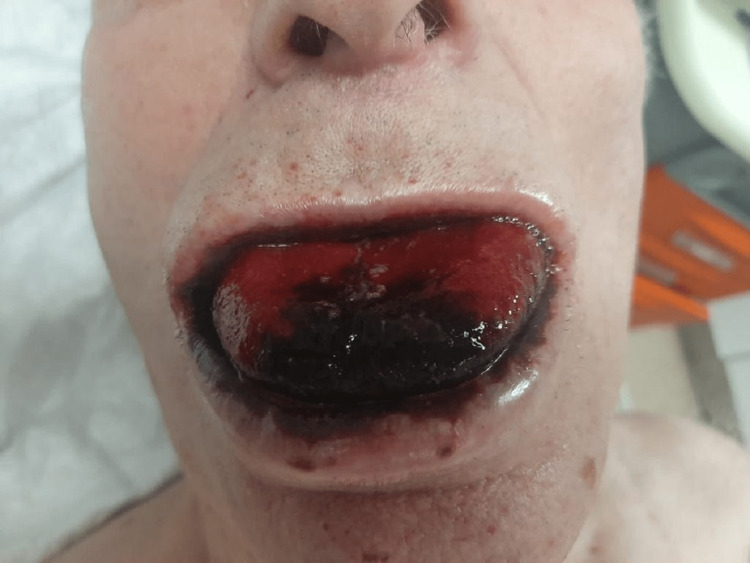
Chemical burns on the oral mucosa and tongue at admission to the emergency room.

An 83-year-old man, with a clinical frailty scale of four, with no relevant medical history, namely no history of depression, psychiatric disease, or previous suicide attempts, is admitted to the emergency room after intentionally ingesting about 200mL of bleach in gel formulation. On admission, the patient was awake, with extensive burns on the tongue (Figure 1), oral mucosa, lips, and blood in the oral cavity. Despite presenting signs of respiratory distress, such as the use of accessory muscles and tachypnea, he had a normal lung auscultation with a peripheral oxygen saturation of 95%, no signs of circulatory shock, and without analytical changes. Due to the risk of airway compromise, orotracheal intubation guided by video laryngoscopy was decided with no complications. During the procedure, edema of the epiglottis and vocal cords was identified. A cervico-thoraco-abdominal computed tomography was performed without signs of perforation or stricture. He was then admitted to the Intensive Care Unit (ICU).

**Figure 2:**
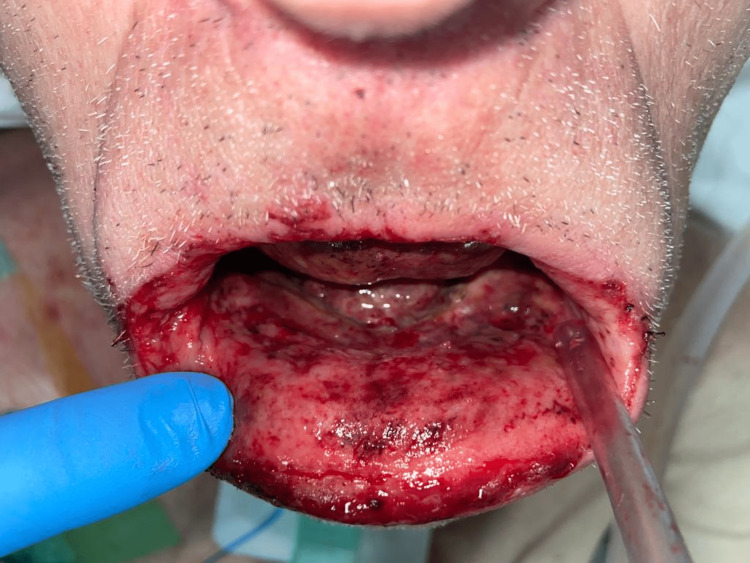
Injury of the oral mucosa a week after admission

The patient presented an initial unfavorable evolution during hospitalization in the ICU despite the absence of signs of superinfection, respiratory failure, or multiorgan dysfunction. Extensive necrosis was observed affecting the entire oral cavity (Figure 2), from the sublingual region to the retromolar triangle, pharyngeal velum, and posterior wall, with loss of mucosal permeability and esophageal injury by caustic grade III (Zargar classification) confirmed by endoscopy.

He was intubated and ventilated for six days and hospitalized in the ICU for one month. Although presenting significant morbidity, the patient was stable enough to be transferred to the general surgery ward. A Stamm gastrostomy was performed, followed by the placement of a percutaneous endoscopic gastrostomy due to esophageal stricture resulting from caustic ingestion. He was discharged after 23 days.

Three months later, in the follow-up consultation, he presented with a clinical frailty scale of seven, an inability to manage sialorrhea, and showed symptoms of depression and anxiety. It was also documented that he was a high family burden due to all the psychological and physical challenges experienced by his family as a consequence of the illness. They were referred to a psychology consultation.

## Discussion

Although limited, caustic intoxication data estimate that ingesting more than 150mL of diluted solutions is likely to cause multiorgan dysfunction [9]. The clinical approach of these patients should involve a careful anamnesis to determine the type and amount of substance ingested. Also mandatory is an objective examination to evaluate the airways, with recognition and treatment of circulatory shock and adequate stabilization. In prehospital care, immediate dilution with a glass of water is recommended. Gastric lavage and activated charcoal are contraindicated unless there is an extreme suspicion of co-ingestion with another toxic substance.

The priority in these cases is airway management - which is considered difficult and must be addressed by an expert. A cardinal sign of ingestion of an alkaline agent is the presence of chemical burns on the mucosa of the oral cavity [10], as seen in this patient. Some other presenting features include dysphagia and sialorrhea.

Esophagogastroduodenoscopy is an essential diagnostic tool in evaluating caustic injuries within 48 hours to classify the extent of the injuries [11], and surgical intervention is recommended in case of perforation [11]. This is considered the cornerstone in the diagnosis, prognostication, and guide for managing caustic ingestions [12].

Mortality after a caustic ingestion is high worldwide and can be up to 20% [6]. Zargar et al. [12] found that early major complications and death were confined to patients with grade III injuries. Therefore, given the severity of the injuries, the age and functional status of the patient, and the numerous and severe post-corrosive complications of the upper gastrointestinal tract described in the literature [13], conservative measures were decided in a multidisciplinary team discussion.

Despite surviving, the patient presented a slow evolution, with the need for surgical interventions post-stabilization and associated extreme physical and psychological morbidity. Additionally, due to the patient's state and increased dependency, signs of caregiver burnout were also observed in family members. For this reason, a psychology consultation was requested. The follow-up consultation has a fundamental role in the reassessment of the patient and his family to guide the best possible quality of life.

## Conclusions

Despite all that is known about the epidemiology and pathophysiology of caustic ingestion, medical teams have difficulty dealing with the injuries and complications created by this problem. This is due not only to the wide range of ages that can be affected but also to the variety of symptoms, severity of the lesions, and their long-term evolution. Although the available treatment options seek to treat immediate injuries and prevent later complications, the individual assessment of each patient, considering their age, frailty, background, and the evaluation of the long-term prognosis, should be a priority.
